# Aluminium Acetate Solution in the Treatment of Wounds

**Published:** 1910-08-27

**Authors:** 


### ALUMINIUM ACETATE SOLUTION IN THE TREATMENT OF WOUNDS.

Some very useful suggestions as to the treatment of certain common surgical conditions were made by Mr. H. F. Waterhouse in an address before the Annual Meeting of the Sevenoaks Division of the British Medical Association. Among other things that he referred to was the great usefulness of aluminium acetate solution in the treatment of wounds.

Burns are treated in his wards with dressings wetted with a 1 per cent. solution of aluminium acetate, and for certain surgical purposes this is advocated as one of the best antiseptic solutions, though it is unknown to most surgeons and practitioners. It is suggested that one of the reasons for neglect of so good a remedy is that it finds no place in the British Pharmacopœia. Mr. Waterhouse states with assurance that the 1 per cent. solution of aluminium acetate is an admirable antiseptic which at the same time is non-toxic, non-irritant, and yet markedly astringent. It is not to be employed in surgical operations, as it spoils steel instruments; but as an antiseptic for moist fomentation of wounds that are infected or probably unclean, or as a medicament for a bath in which to place an infected hand or foot for continuous irrigation, he recommends it strongly. It is used extensively upon the Continent instead of the comparatively weak boric acid solution so generally employed in this country.

The common strength is that of 1 drachm of the liquor aluminii acetatis of the German Pharmacopœia (a 7½ per cent. solution) to 1 fluid ounce of water. Experience shows that there is no danger of poisoning from it, even when a crushed arm or leg is bathed in the solution for a month, and it is stated with confidence that by the employment of continuous irrigation by means of a bath of the 1 per cent. solution of aluminium acetate, pyogenically infected hands and feet, which but for the action of the solution would have called for amputation, have been saved. For dermatitis whatever its cause, for suppurating open wounds, and for cutaneous erysipelas, much is to be said for the favourable results obtainable with fomentation with a 1 per cent. solution of aluminium acetate. There is one objection that may be mentioned, and that is that after three weeks of continuous irrigation of a member such as the hand the surface tissues may assume a ligneous hardness, so that they can scarcely be cut.

Mr. Waterhouse adds some important remarks upon the circumcision of infants in this connection. It is, as he says, one of the most frequent operations that the practitioner is called upon to perform. He refers to the results obtained, and says that one cannot honestly state that they have always been satisfactory; that there has not very frequently been hæmorrhagic effusion under the sutures, sepsis, or some other troublesome complication. To avoid these he advises that half an hour prior to the operation the entire penis should be painted by the nurse with a 2 per cent. solution of iodine in rectified spirit; as the child is being anæsthetised the organ is repainted. The prepuce is now removed distal to a Kocher's artery forceps applied obliquely from above downwards and forwards; the inner lining of the prepuce, having been slit up along its dorsal aspect, is cut short, only about one-eighth of an inch being left. Two sutures are now inserted; one connects the skin and inner lining in the region of the frænum, the other the same two above on the dorsum of the penis. A Turkey towelling-sponge, four inches square, is now perforated in its centre in order to allow the penis to pass through the opening; this ensures the organ being kept in a fairly vertical position. A couple of feet of double zinc cyanide gauze are now rolled loosely round the incision and are wetted with a 1 per cent. solution of aluminium acetate, the nurse being instructed to keep the dressing wet by moistening it hourly with this solution. It is claimed that if ordinary aseptic precautions are observed during the performance of the operation the common haphazard results would be very much improved, and that in at least 96 per cent. of cases healing would be by first intention.

